# Responses to ethanol in C57BL/6 versus C57BL/6 × 129 hybrid mice

**DOI:** 10.1002/brb3.29

**Published:** 2012-01

**Authors:** Jana P Lim, Mimi E Zou, Patricia H Janak, Robert O Messing

**Affiliations:** Ernest Gallo Clinic and Research Center, Department of Neurology, University of California San FranciscoEmeryville, California 94608

**Keywords:** Alcohol, conditioned place preference, ethanol preference, gene targeting, loss of righting

## Abstract

Although genetic background alters responses to ethanol, there has not yet been a methodical quantification of differences in ethanol-related behaviors between inbred and hybrid mice commonly used in gene-targeting studies. Here, we compared C57BL/6NTac × 129S6/SvEvTac F1 hybrid mice (B6129S6) with C57BL/6NTac inbred mice (B6NT), and C57BL/6J × 129X1/SvJ (B6129X1) and C57BL/6J × 129S4/SvJae F1 hybrids (B6129S4) with C57BL/6J mice (B6J), in five commonly used tests: continuous access two-bottle choice drinking, intermittent limited-access binge drinking, ethanol clearance, ethanol-induced loss of the righting reflex, and conditioned place preference (CPP) for ethanol. We found that inbred B6J and B6NT mice showed greater ethanol preference and consumption than their respective hybrids when ethanol was continuously available. Within the intermittent limited-access drinking procedure, though all lines showed similar intake over eight drinking sessions, the average of all sessions showed that B6NT mice drank significantly more ethanol than B6129S6 mice. In addition, B6J mice consumed more ethanol than B6129X1 mice, although they drank less than B6129S4 mice. No differences in ethanol LORR duration were observed between inbred and hybrid mice. Although ethanol clearance was similar among B6J mice and their respective hybrids, B6NT mice cleared ethanol more rapidly than B6129S6 mice. All lines developed CPP for ethanol. Our findings indicate that it may not be necessary to backcross hybrids to an inbred B6 background to study many ethanol-related behaviors in gene-targeted mice.

## Introduction

C57BL/6 (B6) is the most commonly used mouse strain in neuroscience. Although recently it has become possible to generate gene-targeted mice using embryonic stem (ES) cells derived from B6 mice, most have been made using mouse ES cell lines derived from 129 mouse substrains such as 129S6/SvEvTac (W4 cells), 129X1/SvJ (RW-4 cells), and 129S4/SvJae (J1 cells) (Simpson et al. 1997; Auerbach et al. 2000). Following homologous recombination, 129 ES cells are usually implanted into blastocysts harvested from B6 females to generate chimeric progeny (Brook and Gardner 1997). These chimeras are crossed with B6 mice to determine germline transmission in the B6 × 129 hybrid F1 generation. Chimeras that show germline transmission may be crossed with 129 inbred mice to maintain the mutation on an isogenic 129 line, while heterozygous F1 hybrids can be intercrossed to generate F2 hybrid wild-type and mutant mice for experiments or backcrossed with B6 mice for several generations to generate a congenic B6 line that carries the mutation. Highly backcrossed B6 mice are often desirable because their genetic background is nearly homogeneous and much is known about wild-type B6 phenotypes. However, since backcrossing takes considerable time and resources, inbred lines may express phenotypes that interfere with certain experiments, and inbred lines often yield fewer pups per litter than hybrid mice, studies are often performed using wild-type and mutant hybrid mice of the F2 generation where the contribution of DNA from both genetic backgrounds is ∼50% in all mice. A supply of experimental F2 hybrids can be maintained by intercrossing heterozygous F1 breeders, which are in turn replenished by crossing 129 inbred mutants with wild-type B6 mice.

Besides considerations of time, cost, and litter size, hybrid mice may be more appropriate for studies in which wild-type B6 mice show an extreme phenotype. For example, the genetic background of mice greatly influences their preference and response to ethanol (Bachmanov et al. 1996; Blednov et al. 2005; Yoneyama et al. 2008); B6 mice exhibit a high ethanol preference in many paradigms, including continuous access two-bottle choice and limited access binge drinking (Belknap et al. 1993; Rhodes et al. 2007). Thus, to determine if a mutation increases drinking, it may be best to use B6 × 129 hybrid mice as moderate drinkers to avoid a ceiling effect. Another consideration is the choice of 129 substrain since several behavioral differences have been observed among them (Bothe et al. 2004). Therefore, a methodical analysis of differences between B6 inbred lines and their B6 × 129 hybrid counterparts would be useful for planning new ethanol studies and for interpreting prior studies that used different strains.

Here, we examined five strains of mice: B6NT and B6J inbreds, and B6129S6, B6129X1, and B6129S4 hybrids in five ethanol studies: continuous access two-bottle choice drinking, intermittent limited-access binge drinking, ethanol-induced loss of the righting reflex (LORR), ethanol clearance, and conditioned place preference (CPP) for ethanol. Our findings may inform decisions on whether or not to backcross newly generated hybrid lines of gene-targeted mice to study ethanol-related behaviors.

## Materials and Methods

### Rodent care and breeding

Mice were housed under a 12:12 h light–dark cycle (lights off from 7 PM to 7 AM), except for mice used in the limited-access drinking procedure (see below). A naïve group of mice was used for each experiment, with the exception of the ethanol clearance tests, which were performed on mice that underwent the loss of righting procedure at least one week prior. All mice had ad libitum access to food and water. All experiments were conducted using eight- to 13-week-old male mice. For experiments involving B6129S6 mice, C57BL/6NTac mice obtained from Taconic were compared with C57BL/6NTac × 129S6/SvEvTac (B6129S6) F1 hybrids obtained from Taconic or bred in house by mating C57BL/6NTac females with 129S6/SvEvTac males. No differences in ethanol-related behaviors were observed between mice generated at Taconic versus mice bred in house. For experiments involving B6129X1 and B6129S4 mice, C57BL/6J mice were obtained from The Jackson Laboratory and compared with C57BL/6J × 129X1/SvJ (B6129X1) and C57BL/6J × 129S4/SvJae (B6129S4) F1 hybrids. Both of these hybrid lines were bred in house by mating C57BL/6J females with 129X1/SvJ or 129S4/SvJae males. All procedures were approved and conducted in accordance with Gallo Center Institutional Animal Care and Use Committee policies and NIH guidelines for the care and use of animals in research.

### Continuous access two-bottle choice ethanol drinking

Naïve mice were singly housed for one week and then were given 24-h access to two bottles, one containing ethanol and the other water. The ethanol concentration was increased every four to five days as follows: 3%, 6%, 10%, 14%, and 20% (v/v) ethanol in water. Bottles were weighed on Monday, Wednesday, and Friday, and the positions of the bottles were alternated every day to control for any side preferences. Baseline water consumption was measured prior to the start of the ethanol series, and mice were weighed weekly throughout the study. Ethanol consumption was measured as the difference between bottle weights between weighing sessions and calculated as g ethanol/kg mouse/24 h. Ethanol preference was calculated as g ethanol/g total fluid consumed/24 h. Drinking volumes were corrected for spillage by subtracting loss of liquid from two bottles of ethanol and water placed on empty cages in the same rack as experimental cages. At the end of the ethanol series, mice were given access to two bottles of water for one week. They were then given 24-h access to a bottle of water and a second bottle of water flavored with either saccharin (sweet) or quinine (bitter) for two days to test taste reactivity. These tastants were provided in a series that was as follows: 0.03% saccharin, 0.06% saccharin, 0.015 mM quinine, and 0.03 mM quinine. Saccharin and quinine consumption was measured as the difference in bottle weights between days as gram flavored solution drank/kg mouse/24 h and preference was measured as g flavored solution drank/total solution/24 h. Bottle positions were alternated daily and control bottles were included to correct for spillage.

### Intermittent limited-access drinking

Ethanol-naïve mice were individually housed in a reverse light–dark cycle room (lights off from 10 AM to 10 PM) and allowed to acclimate for two weeks. Following acclimatization, home cage water bottles were replaced with a single bottle of 20% (v/v) ethanol in water 2 h after lights off for 4 h on Monday, Wednesday, and Friday, for a total of eight sessions. Bottles were weighed before and after each session and mice were weighed once per week. Baseline water consumption was measured one day before the beginning of ethanol access by weighing a water bottle before and after a single 4-h session. Mice had ad libitum access to water when ethanol was not present. Ethanol consumption (g ethanol/kg mouse/4 h) was calculated as the difference in bottle weights before and after drinking sessions. Drinking volumes were corrected for spillage by subtracting weight lost from two control bottles of 20% ethanol placed on empty cages for the duration of the sessions. At the end of the eighth and last ethanol access session, 20 μl of blood was obtained from the tail vein of each mouse to measure the blood ethanol concentration (BEC). Blood samples were stored at –80°C until BECs were determined using an NAD-ADH enzymatic assay (Carnicella et al. 2009). This limited-intermittent access procedure leads to high levels of ethanol consumption (7 ± 2 g/kg/4 h) as well as high BECs (>90 mg%) in C57BL/6J mice (Neasta et al. 2010).

### Ethanol clearance

Mice were administered 4.0 g/kg of ethanol i.p. and 20 μl of blood was obtained via tail vein puncture at 30, 60, 90, 120, and 180 min post-injection. BECs were determined using the NAD-ADH enzymatic assay as above.

### Loss of the righting reflex (LORR)

To assess the hypnotic effects of ethanol, mice were administered 3.6 g/kg ethanol *i.p.* and checked for LORR by turning them on their backs. LORR was defined as the inability of the mouse to right itself within 30 sec. Mice were determined to have regained their righting reflex if they were able to right themselves three times within 30 sec. Duration of the LORR was recorded.

### Conditioned place preference (CPP)

CPP for ethanol was measured with a two-chambered apparatus using an unbiased procedure. Mice were trained in a 27.3 × 27.3 cm^2^ Med Associates (St. Albans, VT) open-field apparatus equipped with two chambers that had different floor textures (rods or holes) and wall patterns (vertical or horizontal stripes). A manual guillotine door that was closed during training and open during habituation and test sessions separated the chambers. Prior to training, naïve mice were habituated to the apparatus by injecting them with saline i.p. and then allowing them access to both chambers for 30 min. The following day, half of the mice were administered 2 g/kg ethanol i.p. and placed in one conditioning chamber for 5 min. The next day, they were administered an equivalent volume of saline i.p. and placed in the opposite chamber for 5 min. This two-day pattern was repeated for a total of eight days, resulting in four saline- and four ethanol-conditioning sessions. The other half of the animals received saline on the first, third, fifth, and seventh conditioning day and ethanol on the second, fourth, sixth, and eighth conditioning day. A two-day weekend break occurred after the first four conditioning sessions. Twenty-four hours following the final conditioning session, all mice were injected with saline *i.p.* and allowed access to both chambers for 30 min. The results were analyzed in three different ways. First, the time spent in the ethanol-paired side during the habituation session was subtracted from time spent in that same side during the test session to calculate a CPP score, which was compared to a theoretical mean of 0 (no CPP) and was compared between strains. Second, we subtracted the time spent in the saline-paired side from the time spent in the ethanol-paired side on test day to measure preference for the ethanol-paired side, which was also compared to a theoretical mean of 0 (no CPP) and between strains. Third, we compared the amount of time spent on the rod floor when it was paired with ethanol (rod+) and when it was paired with saline (rod–) for each strain.

### Statistical analyses

All data are shown as mean ± SEM values and were analyzed with Prism 5.0 (GraphPad Software, San Diego, CA). All results were tested for normality using a D’Agostino & Pearson omnibus normality test. For continuous access two-bottle choice ethanol drinking, data were analyzed by two-way ANOVA with ethanol concentration as a repeated measure and mouse strain as a between subjects factor. Intermittent, limited access drinking was analyzed by two-way ANOVA with drinking session as a repeated measure and strain as a between subjects factor. For ethanol clearance, data were analyzed by two-way ANOVA with time as a repeated measure and strain as a between subjects factor. Where there were significant interactions between factors, pairs of means were compared using Bonferroni post-hoc tests. Student's *t*-test was used to analyze LORR data. For CPP, data were analyzed using a one-sample *t*-test or Wilcoxon rank signed test, comparing results with a hypothetical mean or median of 0. Differences between mean or median values were assessed using a two-tailed, unpaired *t*-test, Mann–Whitney test, one-way ANOVA, or two-way ANOVA followed by a Bonferroni post-hoc test, as appropriate. Differences were considered significant if *P* < 0.05.

## Results

### Continuous access ethanol consumption and preference

**Figure 1:**
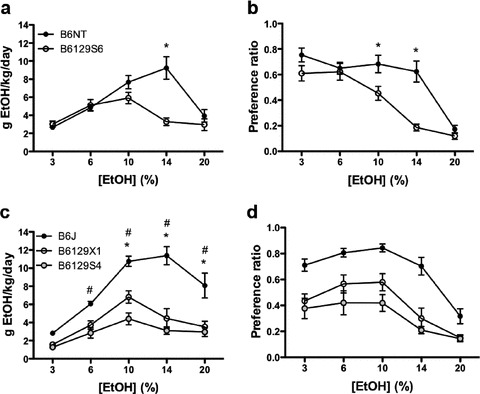
B6129 F1 hybrid mice show decreased voluntary ethanol consumption and preference compared with B6 inbred mice. B6129S6 mice (*n*= 12) showed decreased ethanol consumption (a) and preference (b) when compared with B6NT mice (*n*= 12). *P < 0.05 compared with B6129S6 mice by a Bonferroni post-hoc test. B6129X1 and B6129S4 mice (*n*= 12 per group) showed decreased ethanol consumption (c) and preference (d) compared with B6J mice (*n*= 12). *P < 0.05 for B6129X1 mice compared with B6J, and ^#^*P* < 0.05 for B6129S4 mice compared with B6J by Bonferroni post-hoc tests.

To determine levels of voluntary ethanol consumption and preference, we conducted a continuous access two-bottle choice drinking test. As expected, we found that B6129 mice of all substrains consumed significantly less ethanol than their B6 counterparts. As shown in Figure 1a, hybrid B6129S6 mice consumed less ethanol than B6NT mice [*F*_concentration_(4, 88) = 21.41, *P* < 0.0001; *F*_strain_(1,88) = 6.379, *P*= 0.0193; *F*_concentration × strain_(4, 88) = 12.11, *P* < 0.0001]. They also showed lower ethanol preference [*F*_concentration_ (4, 88) = 51.90, *P* < 0.0001; *F*_strain_(1, 88) = 10.54, *P*= 0.0037; *F*_concentration × strain_(4, 88) = 7.468, *P* < 0.0001]. Post-hoc tests indicated that compared with B6NT mice, B6129S6 mice consumed smaller quantities of 14% ethanol and showed a lower preference for 10% and 14% ethanol.

**Figure 2:**
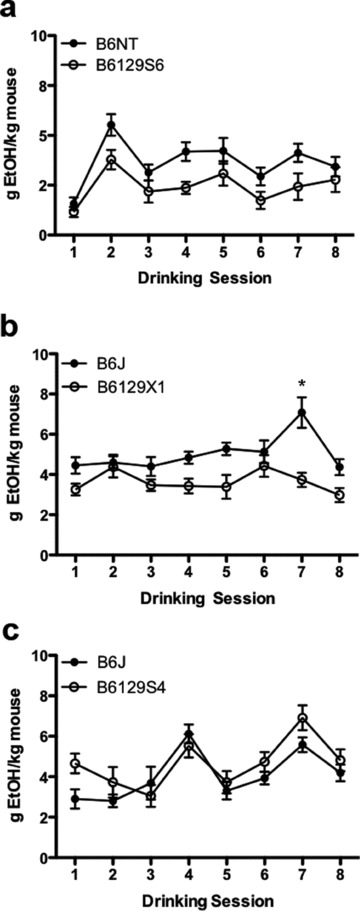
Limited-intermittent access to ethanol drinking in B6129 F1 hybrid and B6 inbred mice. (a) B6129S6 mice (*n*= 10) showed decreased drinking compared with B6NT mice (*n*= 12). (b) B6129X1 mice (*n*= 12) showed decreased drinking on day 7 compared with B6J mice (*n*= 12). *P < 0.05 by a Bonferroni test. (c) B6129S4 mice (*n*= 12) and B6J mice (*n*= 12) consumed similar amounts of ethanol.

When comparing B6J mice with their respective hybrids, we observed qualitatively similar results, although the differences in consumption (Fig. 1c) and preference (Fig. 1d) were present across a greater range of ethanol concentrations. B6129S4 and B6129X1 mice consumed less ethanol than B6J mice [*F*_concentration_(4, 132) = 38.72, *P* < 0.0001; *F*_strain_(2, 132) = 35.94, *P* < 0.0001; *F*_concentration × strain_(8, 132) = 6.099, *P* < 0.0001]. For B6129S4 mice, this difference was present at ethanol concentrations above 3% and for B6129X1 mice at concentrations above 6%. B6129X1 and B6129S4 mice also showed lower ethanol preference than B6J mice (Fig. 2d), with main effects of ethanol concentration [*F*(4, 132) = 34.80, *P* < 0.0001] and mouse strain [*F*(2, 132) = 23.88, *P* < 0.0001], but not a significant interaction between these factors [*F*(8, 132) = 1.74, *P* < 0.09]. Both B6129X1 and B6129S4 hybrid mice showed significantly lower ethanol preference than B6J mice (*P* < 0.01 for both comparisons, Bonferroni test).

We next investigated whether the differences in ethanol preference arose from differences in taste perception between inbred and hybrid strains. We provided mice with water and increasing concentrations of either saccharin (sweet) or quinine (bitter) in a continuous access two-bottle choice test. There were no differences in consumption or preference between B6NT and B6J mice and their hybrid counterparts for saccharin or quinine (Table 1). These data suggest that there are no differences in taste reactivity between the inbred and related hybrid lines.

**Table 1:** Saccharin and quinine consumption and preference are similar in respective hybrid and inbred lines (*n*= 10–12 per group)

	0.03% Saccharin	0.06% Saccharin	0.015 mM Quinine	0.03 mM Quinine
Consumption (g tastant/kg mouse/day)				
B6NT	150.5 ± 10.7	154.7 ± 14.9	39.0 ± 6.9	56.7 ± 8.9
B6129S6	160.1 ± 16.8	200.7 ± 21.8	29.2 ± 6.1	50.6 ± 6.2
B6J	127.9 ± 15.4	190.8 ± 8.8	36.3 ± 6.1	13.7 ± 2.2

B6129X1	118.9 ± 26.6	157.1 ± 18.6	27.0 ± 6.4	9.7 ± 3.7
B6J	148.5 ± 13.2	166.1 ± 23.6	66.3 ± 10.4	28.0 ± 8.1

B6129S4	100.3 ± 15.7	159.1 ± 17.7	44.2 ± 11.02	27. 9 ± 8.5
Preference (g fluid with tastant/g total fluid/day)				
B6NT	0.82 ± 0.03	0.96 ± 0.01	0.34 ± 0.06	0.27 ± 0.04
B6129S6	0.79 ± 0.04	0.92 ± 0.01	0.31 ± 0.05	0.30 ± 0.04
B6J	0.72 ± 0.07	0.94 ± 0.01	0.31 ± 0.07	0.11 ± 0.02

B6129X1	0.56 ± 0.08	0.79 ± 0.06	0.26 ± 0.06	0.08 ± 0.03
B6J	0.86 ± 0.04	0.81 ± 0.09	0.53 ± 0.08	0.23 ± 0.07

B6129S4	0.63 ± 0.09	0.84 ± 0.07	0.40 ± 0.10	0.25 ± 0.08

### Limited-intermittent access binge drinking

To measure binge-like ethanol consumption, we provided mice with limited and intermittent access to ethanol during their circadian dark cycle (Neasta et al. 2010). Using this model, mice achieve levels of ethanol consumption ranging from 3 to 7 g/kg per 4-h session. Comparison of the patterns of drinking across all sessions showed that B6129S6 hybrids consistently consumed less ethanol than B6NT mice with a significant main effect of strain [*F*(1, 140) = 9.34, *P*= 0.006] and session [*F*(7, 140) = 9.66, *P* < 0.001] but no strain by session interaction [*F*(7, 140) = 1.60, *P*= 0.666] (Fig. 2a). B6129X1 hybrids overall also drank less than B6J mice with a significant main effect of strain [*F*(1, 154) = 19.60, *P*= 0.002] and session [*F*(7, 154) = 3.64, *P*= 0.0012] and a strain by session interaction [*F*(7, 154) = 2.51, *P*= 0.0181] that was significant by post-hoc testing at day 7 (Fig. 2b). In contrast, B6129S4 hybrids drank similarly to B6J mice, with no main effect of strain [*F*(1, 153) = 2.46, *P*= 0.131] but a significant effect of session [*F*(7, 153) = 10.60, *P* < 0.001] and no significant strain by session interaction [*F*(7, 153) = 1.49, *P*= 0.176] (Fig. 2c). We also detected these differences when we compared ethanol consumption over all eight binge-drinking sessions; B6129S6 hybrids consumed less ethanol than B6NT mice (*P* < 0.0001) and B6129X1 hybrids less than B6J mice (*P* < 0.0001), but B6129S4 hybrids drank slightly more than B6J mice (*P* < 0.05) (Table 2).

**Table 2:** Average ethanol consumption during limited-intermittent ethanol access.

Mouse Strain	Consumption (g/kg)
B6NT	3.64 ± 0.20
B6129S6	2.44 ± 0.19**
B6J	5.02 ± 0.18
B6129X1	3.63 ± 0.15**
B6J	4.04 ± 0.20
B6129S4	4.65 ± 0.23*

** *P* < 0.0001,

* *P* < 0.05, two-tailed *t*-test; *n*= 10–12 per group.

### Ethanol clearance

**Figure 3:**
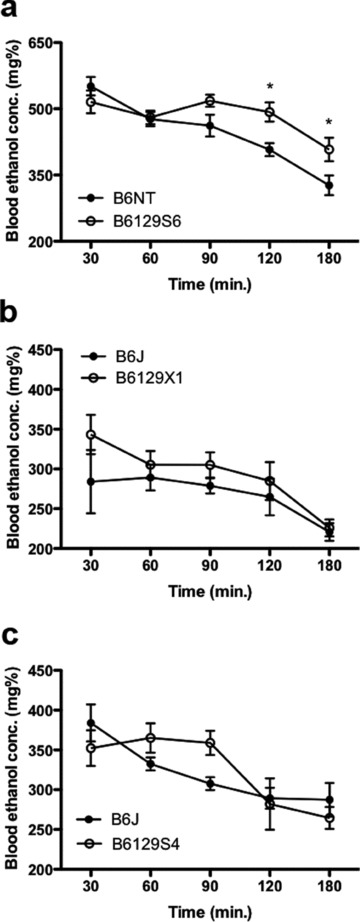
Ethanol clearance rates of B6129 F1 hybrid and B6 inbred mice. (a) B6129S6 mice (*n*= 6) showed decreased ethanol clearance compared with B6NT mice (*n*= 6) at 120 and 180 min postethanol injection (*P < 0.05 by a Bonferroni post-hoc test). (b) B6129X1 and B6J mice showed similar rates of ethanol clearance (*n*= 6 for each strain). (c) B6J and B6NT mice showed similar rates of ethanol clearance (*n*= 6 for each strain).

Since differences in ethanol metabolism could influence ethanol consumption, we measured the rate of ethanol clearance by administering a hypnotic dose (4 g/kg, i.p.) of ethanol and collecting blood samples at several time points thereafter. B6NT mice cleared ethanol more rapidly than B6129S6 hybrid mice [*F*_time_(4, 40) = 18.85, *P* < 0.0001; *F*_strain_(1, 40) = 7.14, *P*= 0.02; *F*_time × strain_(4, 40) = 3.41, *P*= 0.02; Fig. 3a]. For the B6J versus B6129X1 comparison, there was a significant main effect of time [*F*(4, 40) = 6.18, *P*= 0.0006], but not for strain, and no significant strain by time interaction (Fig. 3b). There was a significant main effect of time [*F*(4, 40) = 13.87, *P* < 0.0001] and a strain by time interaction [*F*(4, 40) = 2.80, *P*= 0.04] for the B6J versus B6129S4 comparison, but post-hoc testing did not reveal significant differences between strains at individual time points (Fig. 3c). Comparison of clearance values for B6NT mice (Fig. 3a) with B6J mice (Fig. 3b and c), which were derived from different experiments, suggested that there were differences in ethanol metabolism between these inbred strains. To determine if this represented a true strain difference or was due instead to variation between experiments, we tested B6NT and B6J mice together in one experiment, and found no difference between them in their rate of ethanol clearance (Fig. S1).

### Loss of the righting reflex (LORR)

To examine a behavioral response to a hypnotic dose of ethanol, we examined the duration of the ethanol-induced LORR. There was no difference in LORR duration between B6NT and B6129S6 mice (*P*= 0.18), nor was there a difference between B6J, B6129X1, and B6129S4 mice [*F*(2,29) = 0.06; *P*= 0.94] (Table 3).

**Table 3:** Similar duration of the ethanol-induced loss of the righting reflex in hybrid and inbred lines.

Mouse strain	LORR duration (min)
B6NT	23.40 ± 2.25
B6129S6	28.92 ± 3.10
B6J	23.36 ± 2.62
B6129X1	22.08 ± 2.89
B6129S4	22.44 ± 2.14

*n*= 10–12 per group.

### CPP for ethanol

**Figure 4:**
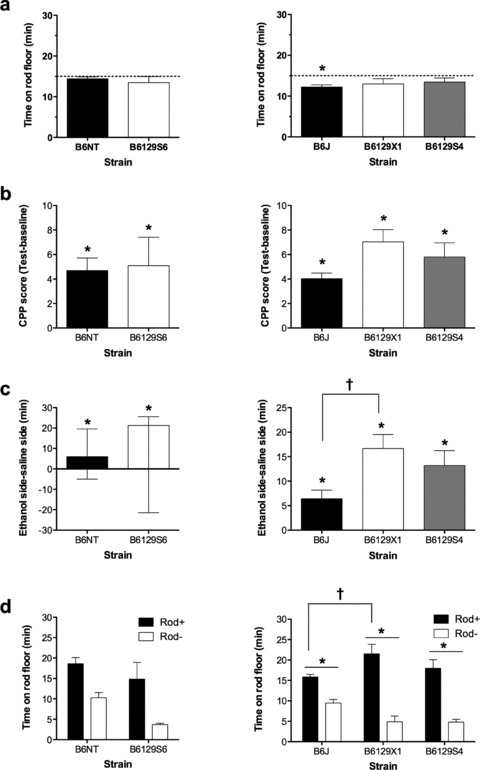
Conditioned place preference (CPP) to ethanol in B6129 hybrid and B6 inbred mice. (a) During baseline habituation, B6J mice spent significantly less time on the rod floor than on the hole floor (*P < 0.05 compared with 15 min, one-sample *t* -test). (b) CPP scores for all strains were significantly greater than 0 (*P < 0.05), indicating the presence of CPP for ethanol. (c) On the test day, all strains showed a preference for the ethanol-paired side (*P < 0.05) and this preference was greater for B6129X1 than for B6J mice (^†^*P* < 0.05). (d) All strains spent more time on the rod floor when it was paired with ethanol than when it was paired with saline. B6129X1 spent more time than B6J mice on the ethanol-paired rod floor (*P < 0.05 and ^†^*P* < 0.05). Data shown are mean ± SEM values except in the left panel in (c), which shows the median and range of data since they were not normally distributed. For (a–c), *n*= 12 per group, and for (d) *n*= 6 per group.

CPP is a widely used procedure to examine the rewarding properties of ethanol and other drugs of abuse. Of all five strains, only B6J showed a small baseline aversion for the rod side of the CPP chamber (Fig. 4a). All strains showed CPP for ethanol when measured by CPP score (Fig. 4b), or preference for the ethanol-paired side on test day (Fig. 4c). There were no significant differences between CPP scores (*P*= 0.88) or preference for the ethanol-paired side on test day (*P*= 0.07) for B6NT strains. After excluding data from one B6J mouse as an outlier by a Grubb's test, we found no significant difference between CPP scores for B6J strains [*F*(2,32) = 2.5, *P*= 0.09]. However, B6129 ×1 mice showed a greater preference for the ethanol-paired side than B6J mice did on the test day [*F*(2,33) = 3.99, *P*= 0.028]. When we analyzed the results by comparing time spent on the rod floor when it was paired with ethanol versus saline (Fig. 4d), we found that both B6NT strains (B6NT, B6129S6) spent more time on the rod floor when it was paired with ethanol (rod+) than when it was paired with saline (rod–) [*F*_pairing_(1,20) = 18.48, *P*= 0.0003], but there was no difference between strains in rod floor pairing with ethanol or saline [*F*_strain × pairing_(1,20) = 0.39, *P*= 0.54]. For B6J strains, there was a significant interaction between strain and floor pairing [*F*_strain × pairing_(2,30) = 5.93, *P*= 0.0068] such that all strains spent more time on the rod floor when it was paired with ethanol than when it was paired with saline, and B6129X1 mice spent more time on the ethanol-paired rod floor than did B6J mice (Fig. 4d).

## Discussion

We investigated whether B6129 hybrid mice show significant differences in ethanol-related phenotypes when compared with their B6 inbred counterparts. We observed the greatest behavioral differences in the continuous access, two-bottle choice test, where hybrid mice consumed significantly less ethanol than inbred mice. In contrast, in the limited access procedure, differences were less pronounced. Indeed, one hybrid line, B6129S4, consumed more than its corresponding inbred line B6J. B6J inbred and hybrid lines showed similar ethanol clearance. In contrast, the comparison of the B6NT mice with B6129S6 mice showed that the hybrid has slower ethanol clearance but only at later time points, 2–3 h following ethanol administration. There was no difference between inbred and hybrid lines in the duration of the LORR. Further, all strains developed a modest CPP for ethanol, with slight differences in magnitude. These results indicate that B6 mice and the related three hybrid lines examined here perform similarly on all behavioral tests except those involving ethanol consumption.

The continuous access two-bottle choice procedure is commonly used to study ethanol self-administration in mice. As expected based on prior studies of ethanol intake in B6 and 129 mice (Belknap et al. 1993; Bachmanov et al. 1996; Yoneyama et al. 2008), we found that B6 mice increased their consumption of ethanol at concentrations that produced a reduction of ethanol consumption and preference in hybrid mice. Hybrid lines showed maximal ethanol consumption with 10% ethanol, while inbred lines peaked at 14%. All lines reduced their ethanol consumption and preference with 20% ethanol, suggesting aversion for this concentration. These results indicate that inbred B6 mice are better suited than B6 × 129 hybrid mice for studying high levels of ethanol consumption in a continuous access procedure. However, hybrid mice may be better suited for detecting the ability of gene disruption to increase drinking in this procedure.

Although the continuous access paradigm can be used to screen for differences in low-to-moderate ethanol consumption, rodents may not consume enough ethanol in this procedure to become intoxicated (Spanagel 2000). To study higher levels of ethanol consumption, investigators have used limited access paradigms providing ethanol in the dark when rodents are more active (Rhodes et al. 2005, 2007; Neasta et al. 2010). In variations of the procedure, mice can consume enough ethanol to reach BECs greater than 80 mg/dL, the level of legal intoxication in many states of the United States (NIAAA 2004). Interestingly, the hybrid strains, which showed low levels of consumption and preference in the continuous access two-bottle choice test at high ethanol concentrations, consumed high levels of 20% ethanol more similar to, or in the case of the B6129S4 mice, slightly greater than their B6 counterparts. This finding demonstrates that the method of ethanol access is important, and challenges the notion of labeling certain mouse strains “ethanol-preferring” and “ethanol non-preferring” without considering the access procedure. This issue should be carefully considered when trying to parse out differences in ethanol drinking between mice of different genetic backgrounds.

Since differences in ethanol metabolism can alter blood ethanol levels, we examined ethanol clearance. The only major difference in clearance we observed was between B6NT and B6129S6 mice at 120 and 180 min postethanol injection, when the hybrids showed slower clearance. The basis for the difference in clearance between these strains is not clear but might relate to strain differences in enzymes that metabolize ethanol and acetaldehyde. B6 mice and certain substrains of 129 mice have different alleles of the gastric isozyme of ethanol dehydrogenase, Adh-3, but the same alleles of the liver isozyme Adh-1 (Holmes et al. 1982). However, this cannot account for the differences in clearance observed here, since ethanol was administered intraperitoneally in this experiment.

To determine if the behavioral response to a hypnotic dose of ethanol was different among the strains, we measured the duration of the LORR. Paralleling the clearance results, the B6129S6 mice displayed a trend toward a longer LORR duration than their B6NT counterparts, although this trend was not statistically significant. Delayed clearance in B6129S6 mice might have contributed to this trend, but recovery from the LORR occurred much earlier (20–30 min) than when the strains showed significant differences in clearance (2–3 h). The development of acute tolerance is a major pharmacodynamic factor that determines recovery from the ethanol-induced LORR (Wallace et al. 2007). Although our clearance and LORR experiments are not entirely comparable since we administered different doses of ethanol (4.0 g/kg for clearance and 3.6 g/kg for LORR), our inability to detect differences in LORR duration might be due to more rapid development of acute tolerance to ethanol in B6129S6 mice compared with B6NT mice, which could dampen the delaying effect of slower ethanol clearance on LORR recovery.

We noted a rather large difference in initial blood alcohol levels at 30 min post-ethanol injection between Taconic and Jackson B6 mice tested in different sessions. To investigate this phenomenon more closely, we compared clearance between B6NT and B6J mice tested together in the same session, and found no difference in either initial blood alcohol levels or clearance rates (Supplemental Fig. 1). We thus concluded that the differences seen between sessions may have been due to variations in environment and timing.

In a final series of experiments, we tested for CPP to ethanol. It has been reported that it is difficult to model CPP to ethanol in B6 mice, with contradictory reports recommending longer and shorter conditioning sessions (Cunningham and Noble 1992; Cunningham, 1995). However, Nocjar et al. (1999) were able to show successful CPP with 16 short, 10-min sessions. Our procedure used a slightly higher ethanol dose and only eight conditioning sessions of 5 min each. This resulted in significant CPP for all lines by three methods of data analysis. Even though B6J mice showed a baseline aversion to the CPP chamber with the rod floor, the effect was small and was unlikely to have been a major influence on our CPP results, even with an unbiased protocol.

Alcoholism in humans is a complex disease that is greatly influenced by genetics, and there are numerous ongoing studies using gene-targeted mice to dissect possible biological pathways. Here, we presented data from a screen of wild-type mice of five different commonly encountered genetic backgrounds. We found that both of the commonly used B6 inbred mouse lines drink considerably more ethanol, and have a greater preference for ethanol when it is continuously available, compared with their respective B6129 F1 hybrids. Hence, if a high level of drinking in a continuous access procedure is desired, it may be advantageous to backcross the transgenic line of interest to a background of greater than 50% B6 relative to 129. However, one may also achieve high levels of drinking even in hybrid mice by using the limited intermittent access procedure described here. For studying other behaviors, it may not be necessary to backcross hybrid mice to generate a congenic B6 line. Our results suggest the importance of considering the genetic background of mice in the design and interpretation of ethanol studies. Importantly, these conclusions also suggest that some ethanol-related behaviors may be tested in newly generated gene-targeted hybrids, thereby saving investigators time and resources involved in backcrossing.

## Supporting Information

**Figure S1.** Ethanol clearance rates for B6NT and B6J inbred mice.

## References

[b1] Auerbach W, Dunmore JH, Fairchild-Huntress V, Fang Q, Auerbach AB, Huszar D, Joyner AL (2000). Establishment and chimera analysis of 129/SvEv- and C57BL/6-derived mouse embryonic stem cell lines. Biotechniques.

[b2] Bachmanov AA, Tordoff MG, Beauchamp GK (1996). Ethanol consumption and taste preferences in C57BL/6ByJ and 129/J mice. Alcohol Clin. Exp. Res.

[b3] Belknap JK, Crabbe JC, Young ER (1993). Voluntary consumption of ethanol in 15 inbred mouse strains. Psychopharmacology.

[b4] Blednov YA, Metten P, Finn DA, Rhodes JS, Bergeson SE, Harris RA, Crabbe JC (2005). Hybrid C57BL/6J x FVB/NJ mice drink more alcohol than do C57BL/6J mice. Alcohol Clin. Exp. Res.

[b5] Bothe GW, Bolivar VJ, Vedder MJ, Geistfeld JG (2004). Genetic and behavioral differences among five inbred mouse strains commonly used in the production of transgenic and knockout mice. Genes. Brain Behav.

[b6] Brook FA, Gardner RL (1997). The origin and efficient derivation of embryonic stem cells in the mouse. Proc. Natl. Acad. Sci. USA.

[b7] Carnicella S, Ahmadiantehrani S, Janak PH, Ron D (2009). GDNF is an endogenous negative regulator of ethanol-mediated reward and of ethanol consumption after a period of abstinence. Alcohol Clin. Exp. Res.

[b8] Cunningham CL (1995). Localization of genes influencing ethanol-induced conditioned place preference and locomotor activity in BXD recombinant inbred mice. Psychopharmacology (Berl).

[b9] Cunningham CL, Noble D (1992). Conditioned activation induced by ethanol: role in sensitization and conditioned place preference. Pharmacol Biochem. Behav.

[b10] Holmes RS, Duley JA, Imai S (1982). Alcohol dehydrogenase isozymes in the mouse: genetic regulation, allelic variation among inbred strains and sex differences of liver and kidney A2 isozyme activity. Anim. Blood Groups Biochem. Genet.

[b11] Neasta J, Ben Hamida S, Yowell Q, Carnicella S, Ron D (2010). Role for mammalian target of rapamycin complex 1 signaling in neuroadaptations underlying alcohol-related disorders. Proc. Natl. Acad. Sci. USA.

[b12] NIAAA (2004).

[b13] Nocjar C, Middaugh LD, Tavernetti M (1999). Ethanol consumption and place-preference conditioning in the alcohol-preferring C57BL/6 mouse: relationship with motor activity patterns. Alcohol Clin. Exp. Res.

[b14] Rhodes JS, Best K, Belknap JK, Finn DA, Crabbe JC (2005). Evaluation of a simple model of ethanol drinking to intoxication in C57BL/6J mice. Physiol. Behav.

[b15] Rhodes JS, Ford MM, Yu CH, Brown LL, Finn DA, Garland T, Crabbe JC (2007). Mouse inbred strain differences in ethanol drinking to intoxication. Genes. Brain Behav.

[b16] Simpson EM, Linder CC, Sargent EE, Davisson MT, Mobraaten LE, Sharp JJ (1997). Genetic variation among 129 substrains and its importance for targeted mutagenesis in mice. Nat. Genet.

[b17] Spanagel R (2000). Recent animal models of alcoholism. Alcohol Res. Health.

[b18] Wallace MJ, Newton PM, Oyasu M, McMahon T, Chou WH, Connolly J, Messing RO (2007). Acute functional tolerance to ethanol mediated by protein kinase C epsilon. Neuropsychopharmacology.

[b19] Yoneyama N, Crabbe JC, Ford MM, Murillo A, Finn DA (2008). Voluntary ethanol consumption in 22 inbred mouse strains. Alcohol.

